# Follow‐Up and Comparative Assessment of SARS‐CoV‐2 IgA, IgG, Neutralizing, and Total Antibody Responses After BNT162b2 or mRNA‐1273 Heterologous Booster Vaccination

**DOI:** 10.1111/irv.13290

**Published:** 2024-05-06

**Authors:** Salma Younes, Eleonora Nicolai, Massimo Pieri, Sergio Bernardini, Hanin I. Daas, Duaa W. Al‐Sadeq, Nadin Younes, Farah M. Shurrab, Parveen B. Nizamuddin, Fathima Humaira, Nader Al‐Dewik, Hadi M. Yassine, Laith J. Abu‐Raddad, Ahmed Ismail, Gheyath K. Nasrallah

**Affiliations:** ^1^ Biomedical Sciences Department, College of Health Sciences Qatar University Doha Qatar; ^2^ Biomedical Research Center Qatar University Doha Qatar; ^3^ Department of Experimental Medicine University of Rome Tor Vergata Rome Italy; ^4^ Clinical Biochemistry Tor Vergata University Hospital Rome Italy; ^5^ College of Dental Medicine, QU Health Qatar University Doha Qatar; ^6^ Department of Basic Medical Sciences, College of Medicine, QU Health Qatar University Doha Qatar; ^7^ Department of Research and Translational and Precision Medicine Research Lab, Women's Wellness and Research Center Hamad Medical Corporation Doha Qatar; ^8^ Genomics and Precision Medicine (GPM), College of Health & Life Science (CHLS) Hamad Bin Khalifa University (HBKU) Doha Qatar; ^9^ Infectious Disease Epidemiology Group, Weill Cornell Medicine–Qatar Cornell University, Qatar Foundation – Education City Doha Qatar; ^10^ World Health Organization Collaborating Centre for Disease Epidemiology Analytics on HIV/AIDS, Sexually Transmitted Infections, and Viral Hepatitis, Weill Cornell Medicine–Qatar Cornell University, Qatar Foundation – Education City Doha Qatar; ^11^ Department of Healthcare Policy and Research, Weill Cornell Medicine Cornell University New York USA; ^12^ Laboratory Section, Medical Commission Department Ministry of Public Health Doha Qatar

**Keywords:** antibodies, anti‐S1‐IgA, anti‐S‐RBD IgG, booster, COVID‐19, immune response, neutralizing antibody, vaccination

## Abstract

**Background:**

Priming with ChAdOx1 followed by heterologous boosting is considered in several countries. Nevertheless, analyses comparing the immunogenicity of heterologous booster to homologous primary vaccination regimens and natural infection are lacking. In this study, we aimed to conduct a comparative assessment of the immunogenicity between homologous primary vaccination regimens and heterologous prime‐boost vaccination using BNT162b2 or mRNA‐1273.

**Methods:**

We matched vaccinated naïve (VN) individuals (*n* = 673) with partial vaccination (*n* = 64), primary vaccination (*n* = 590), and primary series plus mRNA vaccine heterologous booster (*n* = 19) with unvaccinated naturally infected (NI) individuals with a documented primary SARS‐CoV‐2 infection (*n* = 206). We measured the levels of neutralizing total antibodies (NTAbs), total antibodies (TAbs), anti‐S‐RBD IgG, and anti‐S1 IgA titers.

**Results:**

Homologous primary vaccination with ChAdOx1 not only showed less potent NTAb, TAb, anti‐S‐RBD IgG, and anti‐S1 IgA immune responses compared to primary BNT162b2 or mRNA‐1273 vaccination regimens (*p* < 0.05) but also showed ~3‐fold less anti‐S1 IgA response compared to infection‐induced immunity (*p* < 0.001). Nevertheless, a heterologous booster led to an increase of ~12 times in the immune response when compared to two consecutive homologous ChAdOx1 immunizations. Furthermore, correlation analyses revealed that both anti‐S‐RBD IgG and anti‐S1 IgA significantly contributed to virus neutralization among NI individuals, particularly in symptomatic and pauci‐symptomatic individuals, whereas among VN individuals, anti‐S‐RBD IgG was the main contributor to virus neutralization.

**Conclusion:**

The results emphasize the potential benefit of using heterologous mRNA boosters to increase antibody levels and neutralizing capacity particularly in patients who received primary vaccination with ChAdOx1.

## 1 Introduction

SARS‐CoV‐2 virus has infected over 800 million individuals, resulting in more than 6.5 million COVID‐19‐related deaths, as of December 10, 2023 [1]. It is crucial to acknowledge that these statistics may underestimate the actual impact due to the absence of reported cases from self‐testing. In response, global mass vaccination campaigns have been initiated, with over 13.33 billion vaccine doses administered to date [2]. The Oxford–AstraZeneca vector‐based vaccine (ChAdOx1) and the mRNA vaccines Pfizer–BioNTech (BNT162b2) and Moderna (mRNA‐1273) are authorized for use in homologous dual‐dose regimens and are extensively used in Europe and the United States [3].

Since the introduction of these three vaccines, evidence has shown that their effectiveness declines over time, especially against milder disease [4, 5, 6, 7, 8]. They are also less effective against the omicron SARS‐CoV‐2 variant compared to earlier variants [9, 10], and a third (booster) dose restores high effectiveness against severe disease [9, 11, 12, 13]. Furthermore, intermittent supply shortages of vaccines, adverse events of vector‐based vaccines, and emerging SARS‐CoV‐2 variants have led to consideration of heterologous regimens (mix‐and‐match vaccination approach). Heterologous combination of vector vaccines followed by boosting with either of the two mRNA vaccines are recommended in some parts of Europe, including Germany [14].

Although limited data are available on the immunogenicity and efficacy of heterologous strategies, they have been used in previous vaccine studies, including experimental vaccines towards Ebola virus [15, 16, 17] and human immunodeficiency virus [18, 19]. This has led to the recommendation of a heterologous mRNA booster vaccination in ChAdOx1 vector‐primed individuals, particularly after the recognition of undesirable events, including cerebral venous thrombosis and thrombocytopenia [20, 21].

The COV‐BOOST study has demonstrated that mRNA vaccines provide a robust booster effect with low reactogenicity, regardless of the vaccination administered in the primary course. On the basis of these findings, the UK Joint Committee on Vaccination and Immunization recommended either BNT162b2 or mRNA‐1273 to be provided as a booster dose no sooner than 6 months after completion of the primary vaccine course [22, 23]. However, the evidence of effectiveness and immunogenicity to support the application of heterologous regimens remains insufficient. The relative degree of antibody response provided by boosted regimens in terms of neutralizing capacity compared to the immune responses induced by natural infection is still unclear. Therefore, this study aims to prospectively enroll two matched cohorts, comprising vaccinated naïve (VN) and naturally infected (NI) individuals, to study the immunogenicity of two mRNA‐heterologous vaccination regimens. We comprehensively assessed neutralizing, total, anti‐S‐RBD‐IgG, and anti‐S1 IgA antibody responses.

## 2 Materials and Methods

### 2.1 Ethical Approval

The Qatar University Institutional Review Board (QU‐IRB 1537‐FBA/21) examined and approved this study. Prior to sample collection, participants completed an informed consent form, which included questions about their demographics and any prior diseases they may have had, including COVID‐19 infection. All samples were obtained in an anonymous manner without the use of identifying information.

### 2.2 Study Design and Sample Collection

**FIGURE 1:**
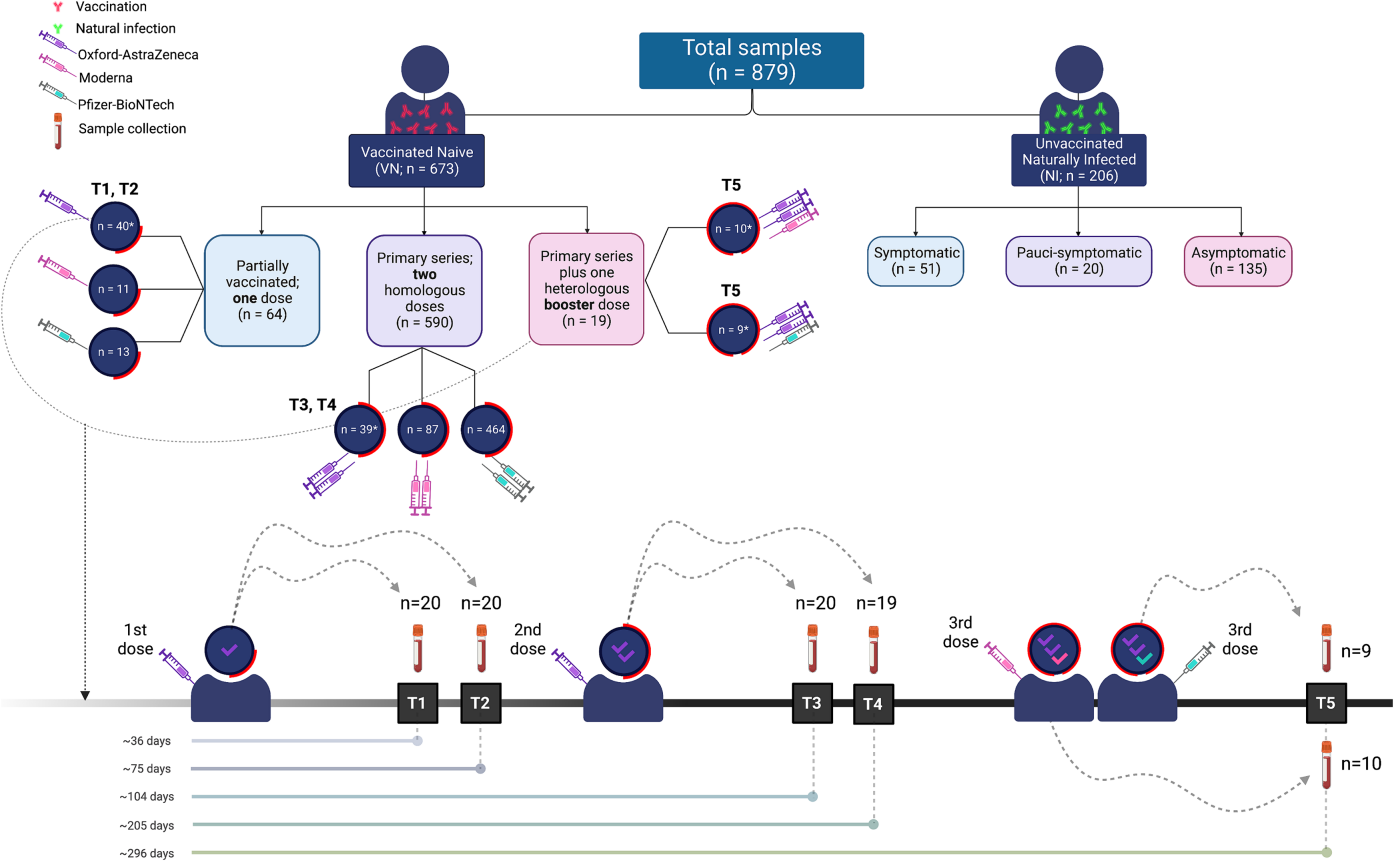
Summary of the study cohort and timeline of sampling. The study included a total of 879 samples. We classified study subjects into two mutually exclusive groups: (1) vaccinated naïve (VN; *n* = 673) and (2) unvaccinated naturally infected (NI; *n* = 206). The VN group was further classified to three subgroups: (1) Partially vaccinated group included samples collected post‐one dose of either ChAdOx1, mRNA‐1273, or BNT162b2. (2) The primary series group included samples collected post–two homologous doses of either ChAdOx1, mRNA‐1273, or BNT162b2. (3) The primary series plus one booster dose group included samples collected post–two doses of ChAdOx1, followed by a heterologous booster shot of either mRNA‐1273 or BNT162b2. * denotes non–mutually exclusive groups, comprising a total of 98 samples collected from 20 VN subjects at five time points (T1–T5). T1 and T2, collected post–first dose, T3 and T4, collected post–homologous second dose, and T5, collected post–heterologous booster (third) dose. Initially, 20 samples from 20 study subjects were collected at each time point; however, two samples at T4 and T5 were excluded due to SARS‐CoV‐2 infection during the follow‐up period. The NI group was further classified to symptomatic, pauci‐symptomatic, and asymptomatic.

The study included a total of 879 samples (Figure 1). We classified study subjects into two main groups: (1) unvaccinated NI (*n* = 206) and (2) VN (*n* = 673).

The NI group (*n* = 206) included samples collected from individuals at median: 67 days post–SARS‐CoV‐2‐confirmed diagnosis. The NI group was further classified according to clinical manifestations into symptomatic (*n* = 51), pauci‐symptomatic (n = 20), and asymptomatic (*n* = 135) (Figure 1).

The VN group (*n* = 673) included samples collected from vaccinated subjects (~105 days from first dose) who had no previous history of infection and were confirmed to be anti‐N negative. The VN group (*n* = 673) was classified according to the number of doses administered, into three subgroups: partially vaccinated (*n* = 64), primary series (*n* = 590), and primary series plus one booster dose (*n* = 19) (Figure 1). Among each group, samples were further classified according to the type of vaccine received (Figure 1).

Among the 673 samples collected from VN individuals, 98 were paired samples collected from the same study subjects at five different time points (T1–T5). T1 and T2 samples were collected post–first dose (~36 and ~75 days from first dose, respectively), T3 and T4 samples were collected post–homologous second dose (~104 and ~205 days from first dose, respectively), and T5 samples were collected post–heterologous booster (third dose; ~296 days from first dose). Figure 1 illustrates the timeline of sampling.

### 2.3 Serology Testing

After collection and centrifugation of whole blood, plasma samples were separated in order to test for (1) neutralizing antibodies (NTAbs), (2) total antibodies (tAbs), (3) anti‐S‐RBD IgG, and (4) Anti‐S1 IgA. All tests were performed against wild‐type (Wuhan/Washington/Victoria strain) SARS‐CoV‐2 virus.

#### 2.3.1 NTAbs

NTAbs (CL‐900i®, Mindray, China) is a competitive binding chemiluminescent immunoassay for quantifying SARS‐CoV‐2 NTAb that disrupts the interaction between the enzyme‐conjugated ACE2 surface receptor and the receptor binding domain (RBD) (bound to magnetic beads) of the viral spike protein. The samples with values over the specified range were diluted with phosphate‐buffered saline (PBS). The WHO conversion factor for the test is 1 AU = 3.31 IU/mL, and the reference range is 10–400 AU/mL. We recently evaluated this new assay and reported that it has great specificity and sensitivity in comparison to two reference techniques [24].

#### 2.3.2 TAbs Against SARS‐CoV‐2 S‐RBD of the SARS‐CoV‐2

The CL‐900i® assay (Catalog No. SARS‐CoV‐2 Total 91 Antibodies 122, Mindray, China) was used to quantify TAbs, comprising IgG, IgA, and IgM. The assay had a positive cutoff index of ≥10–2000 AU/mL. Samples with readings above the range were diluted using PBS.

#### 2.3.3 Antibodies Against the RBD of the S1 Subunit of the Viral Spike Protein (Anti‐S‐RBD IgG)

Antibodies against the viral spike protein's RBD subunit (anti‐S‐RBD) were measured using the quantitative automated platform CL‐900i® (Mindray, China). This assay has a range of 3.0–1000.0 AU/mL, with results ≥10.0 AU/mL considered positive for S‐RBD IgG. Samples exceeding 1000.0 AU/mL were diluted and re‐analyzed. Results were standardized to 1.15 BAU/mL using WHO guidelines.

#### 2.3.4 IgA Against a Recombinant S1 Domain of the SARS‐CoV‐2

The Euroimmun Anti‐SARS‐CoV‐2 IgA assay (Euroimmun, Germany; Cat. No. EI 2606‐9601 A) was performed as directed by the manufacturer. The assay identifies antibodies against the S1 subunit of the SARS‐CoV‐2 spike protein. The results were calculated as a ratio of the sample signal to the average signal of the calibrators. The computed ratios were interpreted in accordance with the manufacturer's recommendations. A ratio of < 0.8 was designated negative, ≥ 0.8 to < 1.1 was considered borderline, and ≥ 1.1 was considered positive [25].

#### 2.3.5 IgG Antibodies Against SARS‐CoV‐2 Anti‐Nucleoprotein (Anti‐N)

Architect‐automated chemiluminescent assay (Abbott Laboratories, USA) and Euroimmun ELISA (El 2606‐9601‐2 G) were used to screen samples for past infection by measuring the SARS‐CoV‐2 anti‐N IgG antibodies, given that IgG antibodies generated against the RBD on the spike protein are distinct from IgG antibodies produced against the nucleoprotein of the virus. Therefore, positive anti‐N findings of SARS‐CoV‐2 anti‐N IgG antibodies imply prior exposure to the whole virus [26]; samples with prior infections were eliminated from the VN group.

### 2.4 Statistical Analysis

The statistical analysis was conducted using GraphPad Prism software (version 9.3.1, GraphPad Software, Inc., San Diego, CA, USA). Continuous variables were summarized by median (interquartile range [IQR]) and categorical variables by number (*n*) (percent). The gathered dataset was evaluated for normality using the Shapiro–Wilk normality test. Due to the lack of a normal distribution, nonparametric tests using the Friedman test for pairwise group comparisons and the Kruskal–Wallis test for the differences between independent samples were conducted. In the bar charts, the horizontal bar line represents the median titer and the error bars represent the IQR. Using the Spearman's rank correlation test, the correlation between NTAbs/anti‐S‐RBD IgG and NTAbs/Anti‐S1 IgA levels was analyzed. A scatterplot was used to illustrate the direction, form, and magnitude of correlation. The significance level was set at *p* < 0.05.

## 3 Results

### 3.1 Descriptive Statistics and Participant Characteristics

A total of 879 samples was included in this study, including samples collected from VN (*n* = 673) and NI (*n* = 206) individuals (Table 1 and Figure 1).

**TABLE 1:** Demographic and clinical characteristics of samples collected from the vaccinated naïve (VN) cohort (*n* = 673) and unvaccinated SARS‐CoV‐2 naturally infected (NI) cohort (*n* = 206).

A. Vaccinated naïve (VN) (*n* = 673)																		
	Partially vaccinated (*n* = 64)						Primary series (*n* = 590)						Primary series plus one booster dose (*n* = 19)				Total	
	ChAdOx1^1^ ^a^		mRNA‐1273^1^		BNT162b2^1^		ChAdOx1^1,2^ ^a^		mRNA‐1273^1,2^		BNT162b2^1,2^		ChAdOx1^1,2^ ^a^ + mRNA‐1273^3^		ChAdOx1^1,2^ ^a^ + BNT162b2^3^			
	(*n* = 40)		(*n* = 11)		(*n* = 13)		(*n* = 39)		(*n* = 87)		(*n* = 464)		(*n* = 10)		(*n* = 9)		(*n* = 673)	
Median age (IQR)	57	55–59	22	20–38	25	21–40	56	54–59	23	20–34	34	22–45	55	53–56	59	57–60	36	22–49
Gender																		
Male, *n* (%)	10	25	5	45.5	1	7.7	10	25.6	40	46	216	46.6	3	30	1	11.1	286	42.5
Female, *n* (%)	30	75	6	54.5	9	69.2	29	74.4	47	54	222	47.8	7	70	8	88.9	358	53.2
Unspecified, *n* (%)	0	0	0	0	3	23.1	0	0	0	0	26	5.6	0	0	0	0	29	4.3
No. of days after administration of 1st dose, median (IQR)	46	36–75	19	16–24	19	15–37	111	104–205	89	60–165	124	68–190	311	282–321	286	279–296	105	61–186
No. of months after administration of 1st dose, median (IQR)	1.5	1.18–2.47	0.63	0.53–0.80	0.63	0.50–1.23	3.65	3.42–6.74	2.97	2.00–5.50	4.12	2.25–6.32	10.21	9.27–10.55	9.4	9.17–9.73	3.5	2.03–6.20

B. Unvaccinated naturally infected (NI) (*n* = 206)								
	Symptomatic		Pauci‐symptomatic		Asymptomatic		Total	
	(*n* = 51)		(*n* = 20)		(*n* = 135)		(*n* = 206)	
Median age (IQR)	33	28–40	27	13–35	37	31–46	36	29–43
Gender								
Male, *n* (%)	33	64.7	14	70.0	131	97.0	178	86.4
Female, *n* (%)	18	35.3	6	30.0	4	3.0	28	13.6
Unspecified, *n* (%)	0	0	0	0.0	0	0.0	0	0.0
No. of days post–COVID‐19 infection, median (IQR)	63	44–146	63	43–149	70	48–86	67	45–98
No. of months post–COVID‐19 infection, median (IQR)	2.07	1.43–4.80	2.05	1.40–4.85	2.3	1.57–2.83	2.2	1.50–3.23

*Note:* ^1^ denotes sample collection post‐partial vaccination, that is, sample collected post–one dose of the indicated vaccine type. ^2^ denotes sample collection post–primary vaccination, that is, sample collected post‐receiving a second dose of the indicated vaccine type. ^3^ denotes a primary series plus one booster dose vaccination, that is, sample collected post–booster dose of the indicated vaccine type.

^a^ Non–mutually exclusive groups, comprising a total of 98 paired blood samples collected from 20 individuals at five time points (T1–T5). TI and T2 included samples collected postadministration of the first dose of ChAdOx1 (ChAdOx1^1^) at median 36 days (IQR: 35–38) and median 75 days (IQR: 72–76) from the first dose. T3 and T4 included samples collected post–second dose of ChAdOx1 (ChAdOx1^1,2^), at median 104 days (IQR: 102–110) and median 205 days (IQR: 200–220) from the first dose. T5 included samples collected post–third dose of either mRNA‐1273 (ChAdOx1^1,2^‐mRNA‐1273^3^) or BNT162b2 (*ChAdOx1^1,2^‐BNT162b2^3^), at median 296 days (IQR: 279–312) from the first dose (Figure 1).

In the VN group, samples were collected at median: 105 days (~3.5 months) after receiving the first dose of either BNT162b2, mRNA‐1273, or ChAdOx1 vaccines. The VN group comprised 42.5% females, 53.2% males, and 4.3% of unspecified gender.

Among the VN group (*n* = 673), the partially vaccinated group (*n* = 64) included samples collected post–one dose of either ChAdOx1 (*n* = 40; 62.5%), mRNA‐1273 (*n* = 11; 17.2%), or BNT162b2 (*n* = 13; 20.3%), hereafter referred to as ChAdOx1^1^, mRNA‐1273^1^, and BNT162b2^1^ VN subjects, respectively. The primary series group (*n* = 590) included samples collected post–two homologous doses of either ChAdOx1 (*n* = 39; 6.6%), mRNA‐1273 (*n* = 87; 14.8%), or BNT162b2 (*n* = 464; 78.6%), hereafter referred to as ChAdOx1^1,2^, mRNA‐1273^1,2^, and BNT162b2^1,2^ VN subjects, respectively. The primary series plus one booster dose group (*n* = 19) included samples collected post–two homologous doses of ChAdOx1, followed by a heterologous booster shot of either mRNA‐1273 (*n* = 10; 52.6%) or BNT162b2 (*n* = 9; 47.4), hereafter referred to as ChAdOx1^1,2^ + mRNA‐1273^3^ and ChAdOx1^1,2^ + BNT162b2^3^ VN subjects, respectively.

Samples were collected from individuals in the NI group at a median of approximately 2.2 months (67 days) after SARS‐CoV‐2 infection. The NI group consisted of 86.4% males and 13.6% females, as indicated in Table 1. Out of the 206 individuals in the NI group, 51 were symptomatic (24.8%), 20 were pauci‐symptomatic (9.7%), and 135 were asymptomatic (65.5%) (Table 1 and Figure 1). The study groups' characteristics, including median age, sex, and median time of blood collection in days, are presented in Table 1.

### 3.2 Heterologous mRNA Vaccine Booster Induced Strong Antibody Responses

**FIGURE 2:**
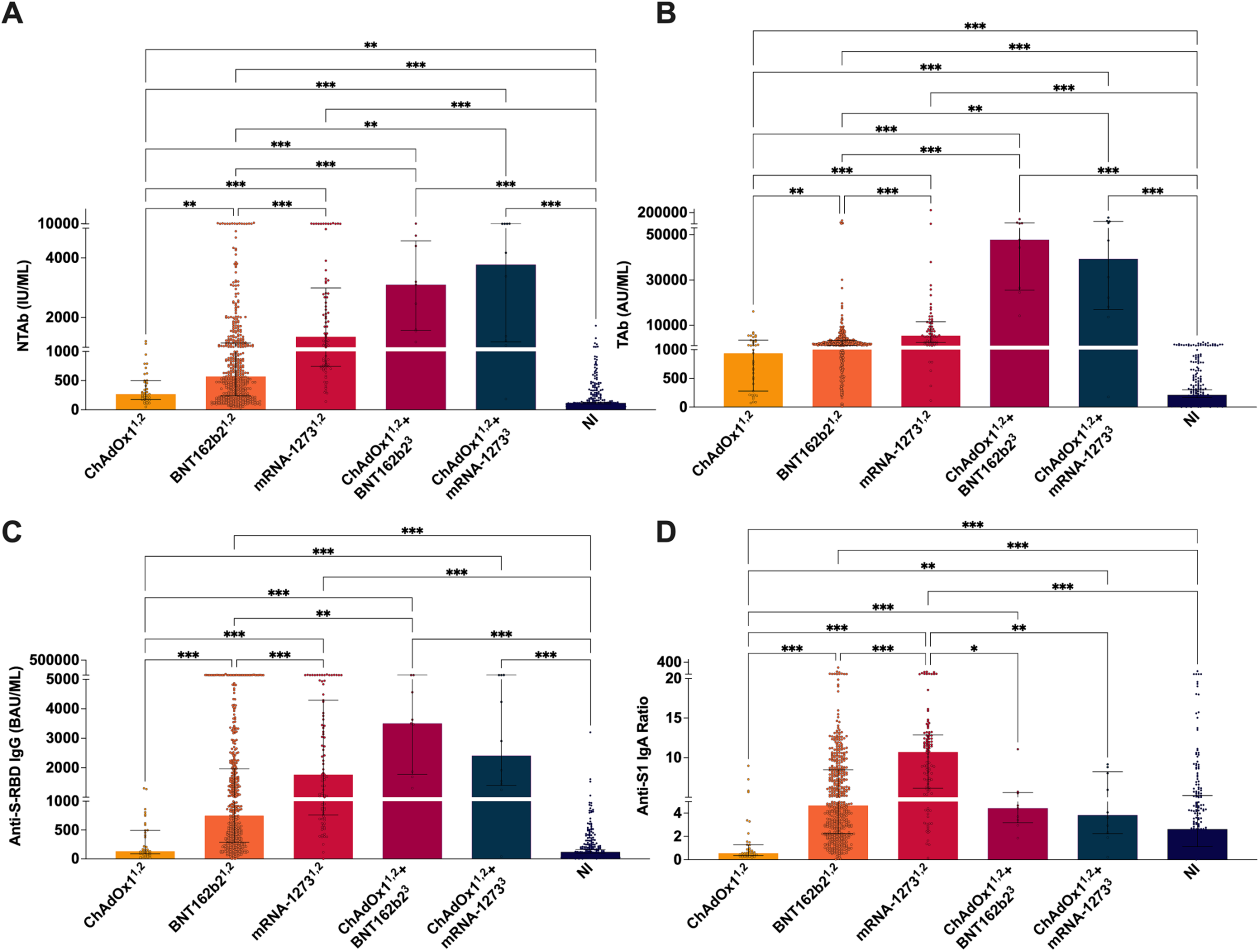
Assessment of vaccine‐induced immunity after heterologous booster with mRNA vaccines. (A) NTAb neutralizing total antibody levels measured by CL‐900i® (IU/mL). (B) TAb total antibody levels measured by CL‐900i. (C) Anti‐S‐RBD IgG antibody levels (BAU/mL) measured by CL‐900i®. (D) Anti‐S1 IgA ratios measured by Euroimmun. Each circle represents a single sample. Black bars indicate interquartile range (IQR). Statistical significance was determined using Kruskal–Wallis test. *p* value asterisk denotes to *p ≤ 0.05, **p ≤ 0.01, and ***p ≤ 0.001.

ChAdOx1^1,2^ showed low NTAb, TAb, anti‐S‐RBD IgG, and anti‐S1 IgA immune responses compared to BNT162b2^1,2^ and mRNA‐1273^1,2^ (Figure 2A–D). The median age for ChAdOx1^1,2^‐vaccinated individuals was 56 (IQR 54–59), while it was 23 (IQR 20–34) for mRNA‐1273^1,2^ and 34 (IQR 22–45) for BNT162b2^1,2^ (Table 1). The administration of a heterologous booster dose, of either mRNA‐1273 or BNT162b2, significantly boosted the humoral immune response elicited by ChAdOx1 vaccine by at least ~12, 42, 24, and 7 folds for NTAb, TAb, anti‐S‐RBD IgG, and anti‐S1 IgA, respectively (Figure 2A–D). Individuals who received mRNA‐1273^3^ booster had a median age of 55 (IQR 53–56), while those who received BNT162b2^3^ booster had a median age of 59 (IQR 57–60) (Table 1).

In the heterologous vaccination regimen, no significant difference was observed between ChAdOx1^1,2^ + Pizer^3^ and ChAdOx1^1,2^ + mRNA‐1273^3^ in any of the assessed antibody isotypes (Figure 1). Nevertheless, NTAb antibodies were slightly higher among ChAdOx1^1,2^ + mRNA‐1273^3^ compared to ChAdOx1^1,2^ + Pizer^3^ group (~1.2 folds), whereas TAb, anti‐S‐RBD IgG, and anti‐S1 IgA antibodies were slightly higher (~1.2, 1.5, and 1.1 folds, respectively) among ChAdOx1^1,2^‐Pizer^3^ compared to ChAdOx1^1,2^‐mRNA‐1273^3^ (Figure 2A–D).

### 3.3 Heterogenous Vaccination With Either BNT162b2 or mRNA‐1273 Boosted ChAdOx1 Humoral Immune Response

**FIGURE 3:**
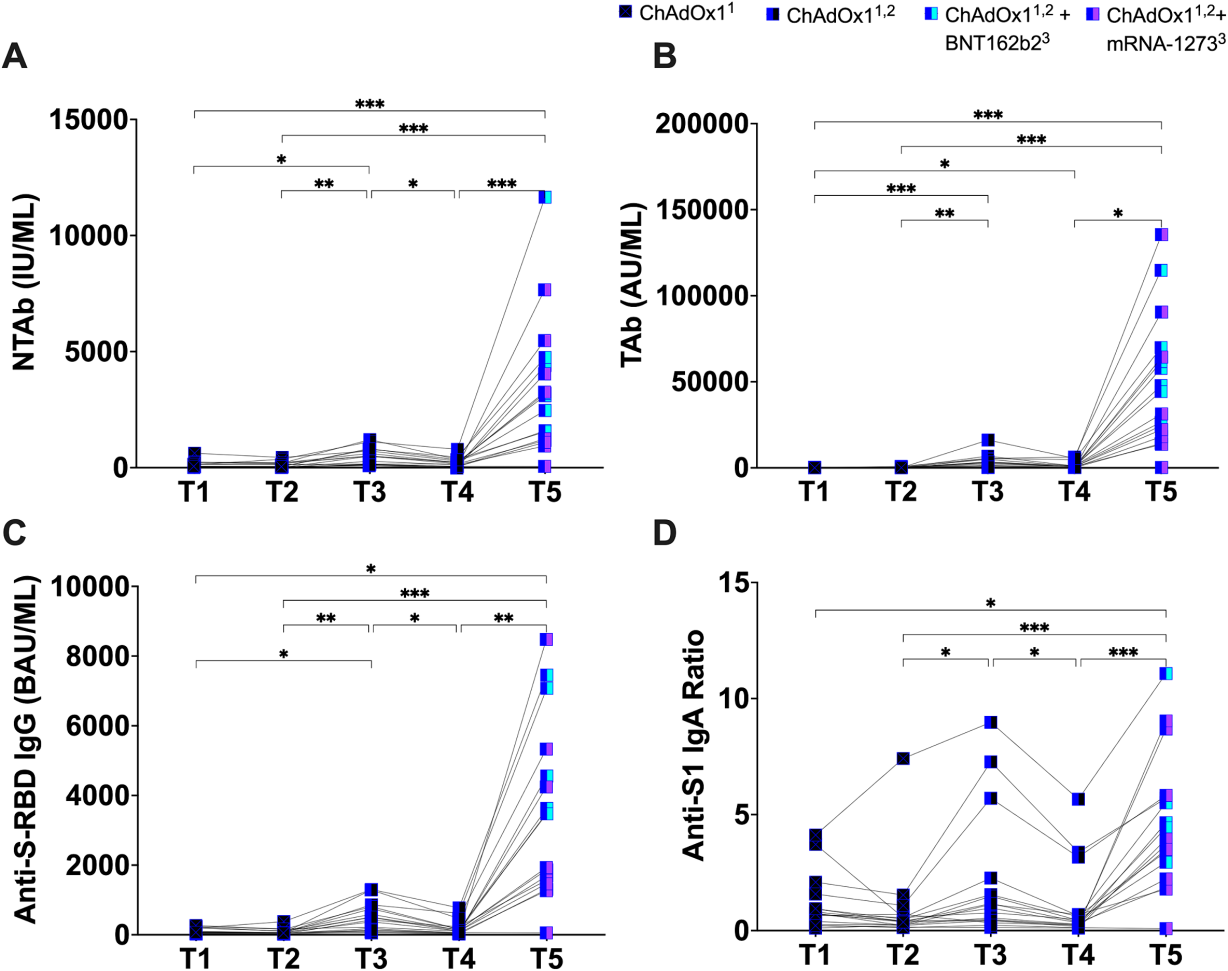
Longitudinal antibody response in VN individuals who received heterologous booster doses. (A) NTAb neutralizing total antibody levels measured by CL‐900i® (IU/mL). (B) TAb total antibody levels measured by CL‐900i. (C) Anti‐S‐RBD IgG antibody levels (BAU/mL) measured by CL‐900i®. (D) Anti‐S1 IgA ratios measured by Euroimmun. A total of 98 samples collected from 20 VN subjects at five time points (T1–T5). T1 and T2, collected post–first dose (~36 and ~75 days from first dose, respectively), T3 and T4, collected post–homologous second dose (~104 and ~205 days from first dose, respectively), and T5, collected post–heterologous booster (third) dose (~296 days from first dose). Initially, 20 samples from 20 study subjects were collected at each time point, however, two samples at T4 and T5 were excluded due to SARS‐CoV‐2 infection during the follow‐up period. The NI group was further classified to symptomatic, pauci‐symptomatic, and asymptomatic. Statistical significance of antibody levels among paired samples was assessed using Friedman test. Mann–Whitney test was conducted for comparisons between T5: ChAdOx1^1,2^ + BNT162b2^3^ and ChAdOx1^1,2^ + mRNA‐1273^3^. *p* value asterisk indicates *p ≤ 0.05, **p ≤ 0.01, and ***p ≤ 0.001. Only significant correlations are shown.

As shown in Figure 3, although a second dose of ChAdOx1 significantly increased antibody response, significant waning in immune responses post–second dose was observed in NTAbs, TAbs, anti‐S‐RBD IgG, and anti‐S1 IgA (Figure 3A–D). In addition, one or two doses of ChAdOx1 vaccine provided far less immune response than those paired with a heterologous BNT162b2 or mRNA‐1273 booster dose following primary series of ChAdOx1 (Figure 3A–D).

### 3.4 Anti‐S‐RBD IgG Contributes More Than IgA to Virus Neutralization Among VN Individuals

**FIGURE 4:**
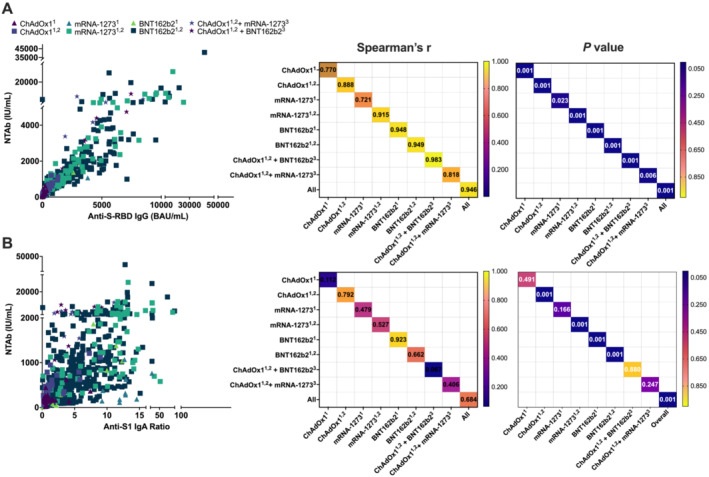
Pairwise correlation of neutralizing total antibody (NTAb) titers with anti‐S‐RBD IgG and anti‐S1 IgA levels among VN individuals. Scatter plots (left) and Spearman's *r* and *p* values' correlation matrices (right) for (A) NTAbs/anti‐S‐RBD IgG and (B) NTAbs/anti‐S1 IgA were generated. Correlation coefficients in the range 0–0.39, 0.40–0.59, 0.6–0.79, and 0.8–1 suggest weak, moderate, high, and very strong correlations, respectively. Scatterplots were used to depict the direction, form, and strength of correlations. All *p* values were two sided at a significance level of 0.05. *p* values < 0.001 is represented as 0.001.

To assess the neutralizing potency and serological dynamics postvaccination, we investigated the correlation between NTAbs/anti‐S‐RBD IgG and NTAbs/anti‐S1 IgA among VN study subjects (Figure 4).

Among the VN group, NTAbs/anti‐S‐RBD IgG showed stronger correlation in comparison to NTAbs/anti‐S1 IgA (Figure 4A,B). The strongest significant correlation (higher Spearman's *r*) was observed for the primary ChAdOx1 series plus one BNT162b2 booster (*r* = 0.983, *p* < 0.001) for NTAbs/anti‐S‐RBD IgG (Figure 4A,B).

Strong to very strong significant correlations between NTAbs and anti‐S‐RBD IgG were observed among individuals of all VN groups (*r* > 0.79, *p* < 0.001) (Figure 4A). In addition, there was a strong significant overall correlation (*r* = 0.684, *p* < 0.001) between NTAbs and anti‐S1 IgA. Nevertheless, stratification by vaccine type and number of dose(s) administered revealed that only partial vaccination with BNT162b2 and primary vaccination with either ChAdOx1, mRNA‐1273, or BNT162b2 showed significant correlations between NTAb and anti‐S1 IgA levels (Figure 4B).

### 3.5 Both Anti‐S‐RBD IgG and Anti‐S1 IgA Significantly Contribute to Virus Neutralization among NI Individuals

**FIGURE 5:**
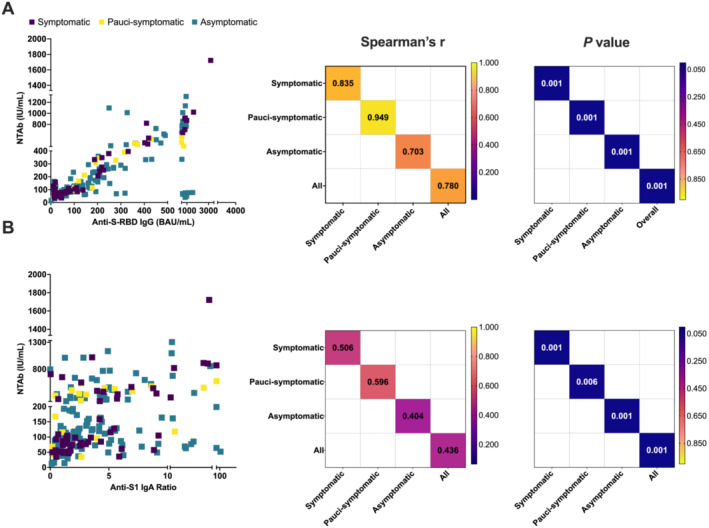
Pairwise correlation of neutralizing total antibody (NTAb) titers with anti‐S‐RBD IgG and anti‐S1 IgA levels among NI individuals. Scatter plots (left) and Spearman's *r* and *p* values' correlation matrices (right) for (A) NTAbs/anti‐S‐RBD IgG and (B) NTAbs/anti‐S1 IgA were generated. Correlation coefficients of 0–0.39 indicate a weak, 0.40–0.59 a moderate, 0.6–0.79 a strong, and 0.8–1 a very strong correlation. Scatterplots were used to depict the direction, form, and strength of correlations. All *p* values were two‐sided at a significance level of 0.05. *p* values < 0.001 is represented as 0.001.

We further sought to assess the serological dynamics and neutralizing potency post–SARS‐CoV‐2 infection. We investigated the correlation between NTAbs/S‐RBD IgG and NTAbs‐IgA among NI (*n* = 206) study subjects (Figure 5A,B).

Interestingly, the NI group showed overall significant correlations between NTAbs and both S‐RBD IgG and anti‐S1 IgA (*p* < 0.001) (Figure 5A,B). However, NTAbs/anti‐S‐RBD IgG showed an overall stronger correlation (*r* = 0.780, *p* < 0.001) in comparison to NTAbs/anti‐S1 IgA, which showed significant but moderate correlations (*r* = 0.436, *p* < 0.001) (Figure 5A,B).

Stratification by clinical manifestations revealed significant correlation between NTAbs/S‐RBD IgG and NTAbs/anti‐S1 IgA among all three groups: symptomatic, asymptomatic, and pauci‐symptomatic (*p* < 0.001). However, the strongest significant correlation (higher Spearman's *r*) was observed for pauci‐symptomatic and symptomatic individuals (*r* = 0.949, *p* < 0.001, and *r* = 0.835, *p* < 0.001, respectively) for NTAbs/anti‐S‐RBD IgG (Figure 4A,B).

## 4 Discussion

In the current study, we aimed to provide a detailed comparative analyses of immunogenicity among heterologous combinations of ChAdOx1 followed by either BNT162b2 or mRNA‐1273 boosting, in comparison to homologous COVID‐19 primary vaccination regimens of BNT162b2, mRNA‐1273, and ChAdOx1. In addition, we compared the observed vaccine‐induced antibody responses to SARS‐CoV‐2 infection‐induced antibody responses.

Our findings revealed considerable differences in the potency and extent of induced humoral immune responses among the assessed vaccination regimens. The most striking finding was that heterologous vaccination with ChAdOx1 followed by either BNT162b2 (*n* = 9, median age: 59, IQR: 57–60) or mRNA‐1273 (*n* = 10, median age: 55, IQR: 53–56) induced robust humoral responses against SARS‐CoV‐2 that are comparable and almost equal to those elicited by homologous BNT162b2 (*n* = 464, median age: 34, IQR: 22–45) and mRNA‐1273 (*n* = 87, median age: 23, IQR: 20–34) primary vaccination regimens alone, but superior to those elicited by homologous ChAdOx1 primary vaccination (*n* = 39, median age: 56, IQR: 54–59) (Table 1, Figure 2A–D). Not only that, but homologous ChAdOx1 vaccination elicited weak antibody responses, with S‐RBD IgG levels being almost equal to those elicited by unvaccinated naturally‐infected individuals (Figure 2C) and S‐RBD IgA being significantly higher among unvaccinated NI individuals compared to ChAdOx1 fully vaccinated individuals (Figure 2D). Furthermore, NTAb antibody responses post–homologous ChAdOx1 vaccination were far less potent compared to homologous BNT162b2 or mRNA‐1273 vaccination (Figure 2A). Similar findings were reported by other studies indicating an overall weaker anti‐spike and anti‐RBD IgG levels among ChAdOx1‐vaccinated individuals compared to mRNA‐1273 or BNT162b2‐vaccinated individuals [27], and that ChAdOx1 in conjunction with mRNA vaccines from Moderna or BioNTech elicited much greater antibody levels than a double dose of ChAdOx1, indicating that mRNA vaccines are the most potent vaccines overall [27].

Despite weak ChAdOx1 immunogenicity, a heterologous booster dose of either BNT162b2 or mRNA‐1273 post–homologous ChAdOx1 vaccination significantly boosted NTAbs, TAbs, anti‐S‐RBD IgG, and anti‐S1 IgA antibodies, by at least ~12, 42, 24, and 7 folds, respectively (Figure 2A–D). It should be highlighted that the profound humoral response elicited by heterologous booster regimens might be attributable to the extended interval between prime and booster dosages. Recent research revealed that with the homologous BNT162b2 vaccination, longer intervals provide greater immunogenicity than the typical 3–4 week interval [28, 29, 30]. Extended booster dosage intervals may result in increased neutralization effect and a broader range of immunologic responses [31]. This aspect may be assessed by comparing immune responses in heterologous immunization to BNT162b2 homologous vaccination at equally extended periods.

Despite the two mRNA heterologous boosters regimens being almost equally potent in inducing humoral antibody response (Figure 2), ChAdOx1/mRNA‐1273 showed higher neutralizing potency (Figure 2A). This is particularly important because the controversy over whether a vaccinated person may spread virus is believed to be influenced in part by their levels of NTAbs. NTAbs are used to prevent infection and to treat SARS‐CoV‐2‐infected patients [32]. In the current study, although a second dose of ChAdOx1 significantly increased NTAbs, that boost was relatively short‐lived, with an observed significant decline in NTAbs ~3 months post–second dose (Figure 3A). Because NTAb levels wane over time postvaccination [8], there is a greater chance that exposure to SARS‐CoV‐2 may result in infection and, thus, COVID‐19 transmission [33].

In this study, it was observed that NTAbs decreased after the second ChAdOx1 immunization (Figure 3), but an mRNA vaccine booster, particularly mRNA‐1273, significantly increased NTAbs by approximately 14 folds, surpassing the levels attained with two homologous ChAdOx1 immunizations. These findings suggest that booster vaccines may not be restricted to matching the vaccines used for the primary series and that vaccine boosters may efficiently raise NTAbs to levels that cannot be attained by primary vaccination regimens [34, 35]. Therefore, multiple‐dose regimen strategies are crucial to maintain high levels of peripheral NTAb, which can limit infection, asymptomatic viral replication, and potential transmission. Although healthcare policies may recommend a third COVID‐19 vaccine at a specific point in time, assessing NTAb levels in vaccine recipients on an individual basis is crucial to determine when an additional dose may be necessary and who may or may not require a third dose. This approach not only preserves vaccines but also avoids vaccinating individuals who already have high levels of NTAbs, as circulating NTAbs may eliminate spike protein as quickly as cells produce it [36].

Although both mRNA booster vaccines demonstrated strong and similar immunogenicity overall, it is important to note that the slight differences in their effectiveness in comparison to primary vaccination regimens could be attributed to several factors. While both vaccines are nucleoside‐modified mRNA vaccines that encode the prefusion stabilized SARS‐CoV‐2 Spike protein, there are differences in their vaccination regimens and formulations [37, 38]. BNT162b2 is given in 100‐μg/mL doses 21 days apart, while mRNA‐1273 is given in 200‐μg/mL doses 28 days apart. Assuming equivalent sized constructions, this means that each mRNA‐1273 dosage generates three times greater Spike protein mRNA copies than BNT162b2, potentially leading to stronger immunogenicity. In addition, certain side effects were more commonly reported after mRNA‐1273 vaccination compared to BNT162b2, and it is possible that this enhanced reactogenicity is accompanied by greater immunogenicity [39, 40]. Furthermore, the nanoparticles utilized to enclose the mRNA in each vaccination are formulated differently, with respect to their lipid content [41].

In the current study, we further determined the contribution of each of the anti‐S‐RBD IgG and anti‐S1 IgA isotypes to virus neutralization among VN (Figure 4) and NI individuals (Figure 5). Our findings revealed that both IgG and IgA significantly contributed to serum neutralization potential among all NI groups, with strongest correlations observed among symptomatic and pauci‐symptomatic compared to asymptomatic individuals (Figure 5). Contrastingly, among VN individuals, anti‐S‐RBD IgG seemed to have a more pronounced contribution more than IgA to serum neutralization potential (Figure 4A). The distinction in isotype contribution between NI and VN individuals may also provide insights into the mechanism of immune memory and protection following natural infection versus vaccination. The substantial role of IgA in NI individuals might reflect mucosal immunity, which is the first line of defense against respiratory pathogens [42], whereas the dominance of IgG in VN individuals could be indicative of the systemic immunity that vaccines aim to establish. This dichotomy underscores the importance of considering both systemic and mucosal immunity in the ongoing development of vaccines and therapeutic strategies.

This research had some limitations. First, the assessment of the antibody response following homologous mRNA vaccination was not feasible due to the absence of data on three doses of the same vaccine type. Additionally, the investigation of antibody responses against different variants was not feasible due to lack of sequencing data and limited sample number. Furthermore, it is important to note that the NI group included only 24.7% symptomatic individuals, which may have influenced the results. The observed antibody responses among the NI group, which predominantly consists of asymptomatic and pauci‐symptomatic individuals, may not fully represent the range of responses seen in individuals with more severe infections [43]. Additionally, the limited number of paired samples in our study poses challenges in establishing direct comparisons and performing in‐depth follow‐up analyses. Despite these limitations, we believe that our study contributes valuable insights into the humoral immune responses associated with different COVID‐19 vaccination regimens.

Despite these limitations, this research has a number of strengths worth consideration. First, the majority of published research has mostly focused on NTAb, IgG, or IgM, but studies on anti‐S1 IgA response are scarce, especially among unvaccinated, NI individuals. Second, in this research, we analyzed anti‐N antibodies, which are essential for identifying people who were infected to a virus but had no symptoms prior to immunization.

## 5 Conclusion

In light of the persistently low COVID‐19 vaccination rates, our study underscores the critical importance of addressing barriers to vaccine uptake. Primary vaccination alone appears to generate substantial antibody levels, but with a limited neutralizing capacity, emphasizing the importance of boosting to achieve robust immunologic responses and maximum protection against SARS‐CoV‐2. However, the declining vaccination rates complicate efforts to achieve the desired level of immunity. Our data demonstrates that administering a heterologous booster dose, of either mRNA‐1273 or BNT162b2, results in a substantial increase in antibody levels and neutralizing capacity. These results strongly support the advantages of administering a third vaccination dosage in containing the SARS‐CoV‐2 pandemic, particularly in light of current concerns regarding the ongoing reluctance to embrace COVID‐19 vaccination. Our study serves as a poignant reminder that, notwithstanding the decline in COVID‐19 cases, the threat endures, and vaccination remains crucial for upholding public health. The ongoing reluctance to vaccination, and the challenges presented by decreasing vaccination rates, highlight the need for targeted interventions and accessible vaccination initiatives. Elevating efforts to foster vaccine acceptance and uptake is a crucial strategy in managing the changing dynamics of the SARS‐CoV‐2 pandemic and averting future waves of infections.

## Author Contributions

**Salma Younes:** Methodology; Writing – original draft; Writing – review and editing. **Eleonora Nicolai:** Data curation; Writing – review and editing. **Massimo Pieri:** Data curation; Writing – review and editing. **Sergio Bernardini:** Data curation; Writing – review and editing. **Hanin I. Daas:** Data curation; Writing – review and editing. **Duaa W. Al‐Sadeq:** Methodology; Writing – review and editing. **Nadin Younes:** Methodology; Writing – review and editing. **Farah M. Shurrab:** Methodology; Writing – review and editing. **Parveen B. Nizamuddin:** Methodology; Writing – review and editing. **Fathima Humaira:** Data curation; Methodology; Writing – review and editing. **Nader Al‐Dewik:** Writing – review and editing. **Hadi M. Yassine:** Writing ‐ review and editing. **Laith J. Abu‐Raddad:** Writing – review and editing. **Ahmed Ismail:** Data curation; Methodology. **Gheyath K. Nasrallah:** Resources; Supervision; Writing – original draft; Writing – review and editing.

## Conflicts of Interest

We would like to declare that all kits used in this paper were provided as in‐kind support for GKN lab.

### Peer Review

The peer review history for this article is available at https://www.webofscience.com/api/gateway/wos/peer‐review/10.1111/irv.13290.

## Data Availability

The data that support the findings of this study are available from the corresponding author upon reasonable request.
